# Development and Validation of a Prediction Model for Survival in Diabetic Patients With Acute Kidney Injury

**DOI:** 10.3389/fendo.2021.737996

**Published:** 2021-12-22

**Authors:** Manqiu Mo, Ling Pan, Zichun Huang, Yuzhen Liang, Yunhua Liao, Ning Xia

**Affiliations:** ^1^ Geriatric Department of Endocrinology, The First Affiliated Hospital of Guangxi Medical University, Nanning, China; ^2^ Department of Nephrology, The First Affiliated Hospital of Guangxi Medical University, Nanning, China; ^3^ Department of Cardiovascular Thoracic Surgery, The Third Affiliated Hospital of Guangxi Medical University: Nanning Second People’s Hospital, Nanning, China; ^4^ Department of Endocrinology, The Second Affiliated Hospital of Guangxi Medical University, Nanning, China

**Keywords:** diabetes, acute kidney injury, prognosis, nomogram, prediction model

## Abstract

**Objective:**

We aimed to analyze the risk factors affecting all-cause mortality in diabetic patients with acute kidney injury (AKI) and to develop and validate a nomogram for predicting the 90-day survival rate of patients.

**Methods:**

Clinical data of diabetic patients with AKI who were diagnosed at The First Affiliated Hospital of Guangxi Medical University from April 30, 2011, to April 30, 2021, were collected. A total of 1,042 patients were randomly divided into a development cohort and a validation cohort at a ratio of 7:3. The primary study endpoint was all-cause death within 90 days of AKI diagnosis. Clinical parameters and demographic characteristics were analyzed using Cox regression to develop a prediction model for survival in diabetic patients with AKI, and a nomogram was then constructed. The concordance index (C-index), receiver operating characteristic curve, and calibration plot were used to evaluate the prediction model.

**Results:**

The development cohort enrolled 730 patients with a median follow-up time of 87 (40–98) days, and 86 patients (11.8%) died during follow-up. The 90-day survival rate was 88.2% (644/730), and the recovery rate for renal function in survivors was 32.9% (212/644). Multivariate analysis showed that advanced age (HR = 1.064, 95% CI = 1.043–1.085), lower pulse pressure (HR = 0.964, 95% CI = 0.951–0.977), stage 3 AKI (HR = 4.803, 95% CI = 1.678–13.750), lower 25-hydroxyvitamin D3 (HR = 0.944, 95% CI = 0.930–0.960), and multiple organ dysfunction syndrome (HR = 2.056, 95% CI = 1.287–3.286) were independent risk factors affecting the all-cause death of diabetic patients with AKI (all *p* < 0.01). The C-indices of the prediction cohort and the validation cohort were 0.880 (95% CI = 0.839–0.921) and 0.798 (95% CI = 0.720–0.876), respectively. The calibration plot of the model showed excellent consistency between the prediction probability and the actual probability.

**Conclusion:**

We developed a new prediction model that has been internally verified to have good discrimination, calibration, and clinical value for predicting the 90-day survival rate of diabetic patients with AKI.

## Introduction

In recent years, the incidence of diabetes has increased globally. According to the International Diabetes Federation Atlas, 9th edition, 463 million adults worldwide live with diabetes as of 2019, with a prevalence rate of approximately 9.3% and an average annual growth rate of 51% (1). Diabetes easily leads to several complications that affect the prognosis of patients with diabetes (2, 3). Approximately 4.2 million people worldwide died from diabetes or its complications in 2019, accounting for approximately 11.3% of all-cause deaths worldwide (1).

Patients with diabetes often develop acute kidney injury (AKI) due to poor blood glucose control, infection, organ failure, contrast agents, and reduced resistance (4, 5). A large retrospective cohort study has shown that the incidence of AKI is 48.6% in diabetic patients, which is significantly higher than that in non-diabetic patients (17.2%) (6). Diabetes can increase the incidence of AKI and the risk of poor renal outcomes (7). Diabetic patients with AKI without timely treatment will progress to chronic renal failure (CRF) and even end-stage renal disease (ESRD), which should be treated by renal replacement therapy (RRT). One study has shown that the RRT rate of AKI in diabetic patients is approximately 5-fold higher than that in non-diabetic patients (8). AKI is not only a common complication of diabetes but also an independent risk factor associated with the survival rate and CRF of diabetic patients (9–11). Diabetic patients with AKI have poorer clinical outcomes (12). Therefore, early identification and intervention of risk factors affecting clinical outcomes can help to delay the progression and improve the survival rate of diabetic patients with AKI.

However, there have been few studies on the factors affecting the prognosis of diabetic patients with AKI. Due to the high prevalence and poor prognosis of AKI in diabetic patients, it is necessary to develop a prognostic model for diabetic patients with AKI. A nomogram is considered a reliable tool that can be used to create a simple intuitive predictive model that quantifies the risk of a clinical event (13, 14). In the present study, an accurate and beneficial prediction model based on a nomogram for predicting the 90-day survival rate of diabetic patients with AKI was developed, aiming to explore the risk factors for poor short-term prognosis and to provide a reference for the prevention and treatment of diabetic patients with AKI.

## Materials and Methods

### Subjects

All subjects were patients treated at The First Affiliated Hospital of Guangxi Medical University from April 30, 2011 to April 30, 2021, who were diagnosed with diabetes and AKI. The inclusion criteria were as follows: 1) a clear diagnosis of diabetes before AKI and 2) changes in serum creatinine (Scr) consistent with the diagnostic criteria for AKI. The exclusion criteria were as follows: 1) age <18 years; 2) patients diagnosed with stage 5 chronic kidney disease (CKD) or who received regular RRT; 3) incomplete baseline data; and 4) patients lost to follow-up within 90 days of AKI diagnosis. The present study was approved by the Ethics Committee of The First Affiliated Hospital of Guangxi Medical University [approval no. 2019(KY-E-028)]. As this was a retrospective analysis of anonymized clinically obtained data and all patient identifiers were removed, there was no need for patients to sign an informed consent form. The present study was conducted in accordance with the tenets of the Declaration of Helsinki (15).

### Research Methods and Groupings

We followed the Transparent Reporting of a Multivariable Prediction Model for Individual Prognosis or Diagnosis (TRIPOD) statement for reporting multivariable prediction model development and validation (16, 17).The TRIPOD checklist of the present study is found in the **Supplementary Material**. A retrospective cohort study was performed, and diabetic patients with AKI were followed up for 90 days or death (death within 90 days). The primary study endpoint was all-cause death within 90 days of AKI diagnosis. Patients were randomly divided into a development cohort and a validation cohort at a ratio of 7:3. The development cohort was used to construct the prediction model, and the validation cohort was used to verify the prediction accuracy of the model.

### Data Collection

Clinical parameters and demographic data were collected, including age, sex, diabetes duration, complications, smoking, drinking, body mass index (BMI), blood pressure, baseline levels of routine blood tests, liver function, renal function, electrolytes, myocardial enzymes, N-terminal prohormone of brain natriuretic peptide (NT-proBNP), 25-hydroxyvitamin D3 [25(OH)D3], AKI stage, infection, heart failure, cerebrocardiovascular diseases, and multiple organ dysfunction syndrome (MODS). The baseline Scr was defined as a stable Scr within the last 3 months or longer if none was available within 3 months (18). ΔScr was calculated as the Scr difference at the end of follow-up and baseline.

### Diagnostic Criteria

The diagnosis of diabetes in our institution complies with the World Health Organization criteria as follows: diabetic symptoms and 1) random blood glucose ≥11.1 mmol/L, 2) fasting blood glucose (FBG) ≥7.0 mmol/L, or 3) postprandial blood glucose (PBG) ≥11.1 mmol/L (19). AKI was diagnosed in accordance with the diagnostic criteria in the guidelines of KDIGO as follows: increase in Scr ≥26.5 μmol/L within 48 h or an increase from the baseline value by ≥50% within 7 days (20). The criteria of AKI stages were as follows: stage 1, AKI was defined by the AKI Network as at least a ≥50% rise or a ≥0.3 mg/dl rise from baseline Scr; stage 2, AKI was defined as a doubling in Scr from baseline; and stage 3, AKI was defined as a tripling in Scr from baseline or receiving acute dialysis during the hospital stay (21). Heart failure complied with the European Society of Cardiology guidelines as follows: the symptoms and/or signs of heart failure with left ventricular ejection fraction less than 40% (22). MODS was defined as acute and potentially reversible dysfunction of two or more organ systems (23).

### Statistical Analysis

Statistical analyses were performed and graphics were produced with SPSS version 22.0 (IBM Corp., Armonk, NY, USA) and R software version 4.0.3 (https://www.r-project.org/). For continuous variables, data are presented as the mean ± standard deviation (normal distribution) or median and interquartile range (abnormal distribution). For dichotomous variables, data are presented as whole numbers and proportions [*n*(%)]. *T*-tests, chi-square tests, or Wilcoxon rank-sum tests were used to compare differences in the clinical data between the development cohort and the validation cohort by SPSS. Univariate Cox regression was used to screen the risk factors affecting the prognosis, and the “forward LR” method was then used to screen the variables of *p* < 0.05 that were included in the multivariate Cox proportional hazards regression model. Based on the results of multivariate Cox analysis, the “rms” package in R statistical software was used to construct the nomogram according to the hazard ratio (HR) and 95% confidence interval (95% CI) of the risk factors. Bootstrapping resampling techniques with 1,000 replications were used to perform internal validation. The concordance index (C-index) and receiver operating characteristic (ROC) curve were used to evaluate the differentiation of the prediction model in the development and validation cohorts. A C-index or area under the ROC curve (AUROC) >0.70 indicated that the prediction effect of the model was good. Calibration plots were drawn to evaluate the accuracy of the prediction model in the development and validation cohorts. The “rms” package was used to draw the calibration plots. Regarding the model, calibration lines closer to the standard line indicate better calibration degree of the model. A *p* < 0.05 was considered statistically significant in all analyses.

## Results

### Characteristics of Patients in the Development and Validation Cohorts

There were 1,254 patients diagnosed with diabetic AKI, of whom 52 were younger than 18 years, 98 received regular RRT, 36 had incomplete baseline data, and 26 were lost to follow-up. Finally, a total of 1,042 patients were enrolled in our study, with 730 and 312 patients assigned to the development and validation cohorts, respectively. In the development cohort, 21.2% (*n* = 155) had stage 1 AKI, 24.4% (*n* = 178) had stage 2 AKI, and 54.4% (*n* = 397) had stage 3 AKI; the median follow-up time was 87 (40–98) days. By the end of follow-up, 86 patients had died (11.8%) within 90 days of AKI diagnosis. The 90-day cumulative survival rate was 88.2% (644/730), and the recovery rate for renal function in survivors was 32.9% (212/644). The main causes of death were cerebrocardiovascular diseases in 37 cases (43.0%), bacterial infection in 28 cases (32.6%), and other or unknown causes in 21 cases (24.4%). In the validation cohort, the median follow-up time was 86.5 (36–99) days. Forty patients died (12.8%) within 90 days of AKI diagnosis. The 90-day cumulative survival rate was 87.2% (272/312), and the recovery rate for renal function in survivors was 33.8% (92/272). The main causes of death were cerebrocardiovascular diseases in 17 cases (42.5%), bacterial infection in 14 cases (35.0%), and other or unknown causes in 9 cases (22.5%).

**Table 1** shows the patient characteristics by cohort. Compared to the validation cohort, patients in the development cohort had higher creatine kinase and lower endogenous creatinine clearance (*p* < 0.05). There was no significant difference in sex, age, diabetes duration, BMI, blood pressure, white blood cell count (WBC), platelets, hemoglobin, 25(OH)D3, creatine kinase-MB (CK-MB), lactate dehydrogenase (LDH), NT-proBNP, FBG, PBG, glycosylated hemoglobin A1c (HbA1c), blood urea nitrogen (BUN), baseline Scr, uric acid (UA), serum kalium levels, incidence of RRT, heart failure, CKD, bacterial infections, or MODS (*p* > 0.05). Kaplan–Meier survival analysis showed that there was no significant difference in the 90-day survival rates between the development and validation cohorts (log rank *χ* ^2^ = 0.208, *p* = 0.648).

**Table 1:** Differences in the development cohort and the validation cohort in terms of demographic characteristics and laboratory values.

Parameters	Development cohort	Validation cohort	*t*/*χ* ^2^/*z*	*p*-value
Male/female	491/239	217/95	0.527	0.468
Age (years)	62.54 ± 13.92	62.32 ± 14.28	0.229	0.819
Diabetes duration (months)	73 (25–124)	75 (27–119)	0.108	0.912
BMI (kg/m^2^)	24.02 ± 4.00	23.93 ± 4.31	0.258	0.796
SBP (mmHg)	133.75 ± 27.33	135.42 ± 25.44	−0.911	0.363
DBP (mmHg)	76.12 ± 16.50	76.23 ± 15.06	−0.101	0.920
PP (mmHg)	57.63 ± 18.67	59.19 ± 18.89	−1.215	0.224
WBC (×10^9^/L)	12.84 ± 7.70	12.78 ± 7.63	0.120	0.905
Hb (g/L)	102.39 ± 23.83	104.47 ± 24.38	−1.220	0.223
PLT (×10^9^/L)	187.65 ± 87.16	194.40 ± 87.19	−1.090	0.276
NEU	0.76 ± 0.16	0.75 ± 0.16	1.002	0.317
Alb (g/L)	31.76 ± 7.56	32.01 ± 7.70	−0.451	0.652
25(OH)D3 (nmol/L)	54.36 ± 23.38	55.96 ± 22.88	−1.018	0.309
CK (U/L)	211 (86–345)	124 (59–351)	−2.881	0.004
CK-MB (U/L)	29.31 ± 26.98	29.25 ± 25.88	0.030	0.976
LDH (U/L)	488.39 ± 496.04	496.29 ± 473.91	−0.223	0.823
NT-proBNP (pg/ml)	5,175.09 ± 3,073.04	5,151.26 ± 3,092.85	0.106	0.915
FBG (mmol/L)	8.42 ± 3.52	8.49 ± 3.46	−0.265	0.791
PBG (mmol/L)	12.44 ± 3.85	12.54 ± 4.12	−0.242	0.809
HbA1c (%)	8.03 ± 2.80	8.26 ± 2.94	−0.946	0.344
BUN (mmol/L)	14.60 ± 9.82	13.35 ± 10.09	1.850	0.065
baseline Scr (μmol/L)	143.08 ± 123.60	136.92 ± 112.34	0.629	0.530
UA (μmol/L)	418.72 ± 198.97	412.70 ± 211.12	0.430	0.667
HCO_3_^−^ (mmol/L)	21.88 ± 5.47	22.05 ± 5.34	−0.440	0.660
Ccr (ml/min)	42.23 ± 25.46	46.38 ± 29.00	−1.991	0.047
Cys-C (mg/L)	2.40 ± 1.48	2.28 ± 1.46	1.163	0.245
ΔScr (μmol/L)	131.59 ± 190.11	115.78 ± 179.67	1.250	0.212
Serum kalium (mmol/L)	4.31 ± 1.75	4.28 ± 0.93	0.234	0.815
RRT, *n* (%)	163 (22.3)	64 (20.5)	0.286	0.593
Bacterial infection, *n* (%)	506 (69.3)	209 (67.0)	0.550	0.458
HF, *n* (%)	257 (35.2)	109 (34.9)	0.007	0.933
CKD, *n* (%)	189 (25.9)	78 (25.0)	0.091	0.763
MODS, *n* (%)	114 (15.6)	50 (16.0)	0.028	0.868
Death, *n* (%)	86 (11.8)	40 (12.8)	0.222	0.637

25(OH)D3, 25-hydroxyvitamin D3; Alb, albumin; BMI, body mass index; BUN, blood urea nitrogen; Ccr, endogenous creatinine clearance rate; CK, creatine kinase; CK-MB, creatine kinase-MB; Cys-C, serum cystatin C; DBP, diastolic blood pressure; FBG, fasting blood glucose; FIB, fibrinogen; Hb, hemoglobin; HbA1c, glycosylated hemoglobin A1c; HF, heart failure; LDH, lactate dehydrogenase; MODS, multiple organ dysfunction syndrome; NEU, neutrophil percentage; NT-proBNP, N-terminal prohormone of brain natriuretic peptide; PBG, postprandial blood glucose; PLT, platelet count; PP, pulse pressure; RRT, renal replacement therapy; SBP, systolic blood pressure; Scr, serum creatinine; UA, uric acid; WBC, white blood cell count; ΔScr, creatinine difference at the end of follow-up therapy.

### Risk Factors Affecting Prognosis

Cox regression analysis was used to construct the prediction model because the Cox proportional hazards assumption was met. As shown in **Table 2**, univariate Cox analysis of the development cohort revealed that advanced age, pulse pressure, WBC, NT-proBNP, ΔScr, AKI stage, serum kalium levels, 25(OH)D3, heart failure, and MODS were related factors for all-cause death of diabetic patients with AKI (*p* < 0.05). Sex, diabetes duration, length of hospital stay, smoking, drinking, BMI, blood pressure, platelets, hemoglobin, CK-MB, LDH, FBG, PBG, HbA1c, BUN, baseline Scr, UA, incidence of proteinuria, RRT, bacterial infection, CKD, coronary heart disease, and cerebrovascular disease were not correlated with death (*p* > 0.05). Hence, these significant indicators [advanced age, pulse pressure, WBC, NT-proBNP, ΔScr, AKI stage, serum kalium levels, 25(OH)D3, heart failure, and MODS] with statistical significance from the univariate analysis were included in the multivariate Cox regression analysis. The results showed that advanced age (every 1 year increase: HR = 1.064, 95% CI = 1.043–1.085, *p* < 0.001), stage 3 AKI (HR = 4.803, 95% CI = 1.678–13.750, *p* = 0.003), and MODS (HR = 2.056, 95% CI = 1.287–3.286, *p* = 0.003) were independent risk factors affecting the all-cause death of diabetic patients with AKI, while higher pulse pressure (every 1 mmHg increase: HR = 0.964, 95% CI = 0.951–0.977, *p* < 0.001) and higher 25(OH)D3 (every 1 nmol/L increase: HR = 0.944, 95% CI = 0.930–0.960, *p* < 0.001) were independent protective factors affecting the all-cause death of diabetic patients with AKI.

**Table 2:** Analysis of risk factors for prognosis in the development cohort (univariate and multivariate Cox regression).

Variables	Univariate		Multivariate	
	HR (95% CI)	*p*-value	HR (95% CI)	*p*-value
Age	1.049 (1.032–1.067)	<0.001	1.064 (1.043–1.085)	<0.001
Pulse pressure	0.984 (0.972–0.996)	0.009	0.964 (0.951–0.977)	<0.001
WBC	1.034 (1.010–1.058)	0.004		
ΔScr	1.002 (1.001–1.003)	<0.001		
AKI stage				
1	1 [Reference]	0.001	1 [Reference]	0.001
2	4.104 (1.687–9.983)	0.002	2.259 (0.769–6.632)	0.138
3	4.833 (2.075–11.259)	<0.001	4.803 (1.678–13.750)	0.003
Serum kalium	1.088 (1.043–1.135)	<0.001		
NT-proBNP	1.001 (1.000–1.001)	<0.001		
25(OH)D3	0.950 (0.937–0.962)	<0.001	0.944 (0.930–0.960)	<0.001
HF	2.272 (1.476–3.497)	<0.001		
MODS	4.178 (2.714–6.432)	<0.001	2.056 (1.287–3.286)	0.003

The forward method was used to screen variables.

25(OH)D3, 25-hydroxyvitamin D3; HF, heart failure; NT-proBNP, N-terminal prohormone of brain natriuretic peptide; WBC, white blood cell count; ΔScr, serum creatinine difference at the end of follow-up and baseline.

### Development and Validation of the Prediction Model

**Figure 1:**
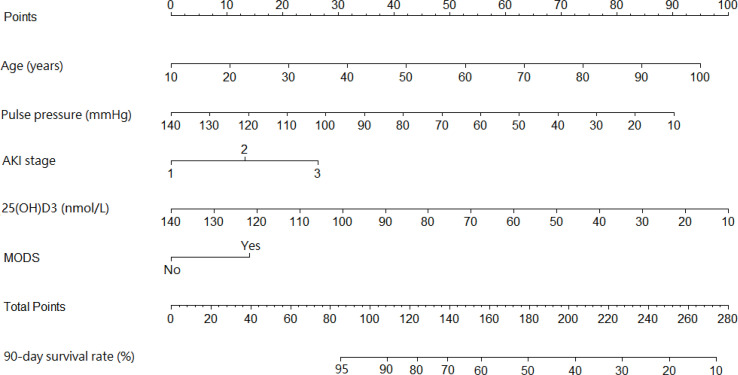
Nomogram predicting the 90-day survival rate in diabetic patients with acute kidney injury (AKI).

A prediction model that incorporated the above independent predictors was developed as the nomogram (**Figure 1**). The 90-day survival rate after the diagnosis of AKI was estimated by calculating risk factor scores. Taking a 65-year-old patient with MODS as an example, the scores of each influencing factor were calculated as follows: 57.5 points for a 65-year-old, 37.5 points for a pulse pressure of 85 mmHg, 26.25 points for stage 3 AKI, 65 points for 25(OH)D3 of 55 nmol/L, and 13.75 points for MODS. The total score of this patient was 200 points (57.5 + 37.5 + 26.25 + 65 + 13.75), and the predicted 90-day survival rate was approximately 42%.

**Figure 2:**
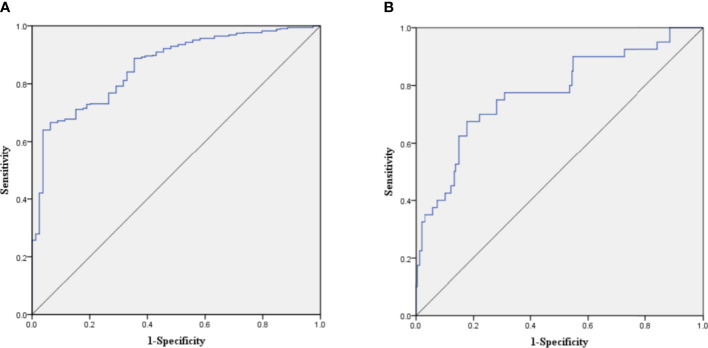
**(A)** Receiver operating characteristic (ROC) curve of the prediction model in the development cohort. **(B)** ROC curve of the prediction model in the validation cohort.

**Figure 3:**
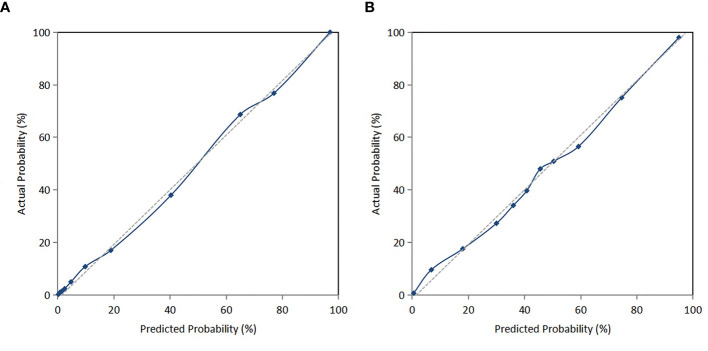
**(A)** Calibration curve of the nomogram in the development cohort. **(B)** Calibration curve of the nomogram in the validation cohort.

The C-index of the prediction model in the development cohort was 0.880 (95% CI = 0.839–0.921). As shown in **Figure 2A**, the AUROC of the prediction model for the 90-day survival rate was 0.860, and the sensitivity and specificity were 0.766 and 0.937, respectively. The C-index of the prediction model in the validation cohort was 0.798 (95% CI = 0.720–0.876) according to the internal verification by Bootstrap. As shown in **Figure 2B**, the AUROC of the prediction model for the 90-day survival rate was 0.774, and the sensitivity and specificity were 0.705 and 0.821, respectively. The risks of death in the decile groups were calibrated using a smoothing function, with the *X*-axis as the predicted probabilities and the *Y*-axis as the actual probabilities. In both the development and validation groups, the calibration plots of the prediction model were close to a straight line with a slope of 1 (**Figures 3A, B** **)**.

## Discussion

The prognosis of diabetic patients with AKI is worse than that of non-diabetic or non-AKI patients (12, 24, 25). Therefore, it is particularly important to explore the risk factors affecting the clinical outcomes and to construct a prognostic model for diabetic patients with AKI. In the present study, the risk factors for short-term prognosis were evaluated using Cox regression analysis, and a prediction model of prognostic risk was constructed based on the clinical parameters and demographic characteristics of diabetic patients with AKI. The results of our study showed that the 90-day survival rate was 88.2%, and advanced age, lower pulse pressure, stage 3 AKI, lower 25(OH)D3, and MODS were independent risk factors affecting the all-cause death of diabetic patients with AKI. Based on these risk factors, a model was established to predict the short-term survival of diabetic patients with AKI. In addition, calibration plots, the C-index of the validation cohort, the AUROC of the validation cohort, and bootstrapping resampling techniques were used for the internal validation of the predictive model. The accuracy verification showed that the model had a certain predictive ability.

Several studies have shown that ketoacidosis, hyperosmolar and hyperglycemic coma, rhabdomyolysis, contrast agents, sepsis, and heart failure are risk factors for the development of AKI in diabetes mellitus (26, 27). If AKI is not corrected in time, the degree of kidney injury might be aggravated. AKI is associated with poor prognosis in patients, including the occurrence of CKD, progression of CKD, prolonged hospital stays, increased adverse cardiovascular events, and mortality (28–31). Previous studies on predictive models of AKI have mainly focused on specific populations of patients with AKI after cardiac surgery (32, 33), AKI after non-cardiac surgery (34, 35), septic AKI (36, 37), tumor-related AKI (38), and critical AKI (39). James et al. (40) constructed a predictive model of progression to advanced CKD after discharge in patients with AKI, and the results showed that older age, female sex, higher baseline Scr, higher baseline proteinuria, more severe AKI, and higher Scr at discharge were associated with a higher risk of progression to advanced CKD. However, few studies have developed or validated prediction models for all-cause mortality in diabetic patients with AKI. To our knowledge, this is the first study to develop a prognostic model for the 90-day survival rate in diabetes with AKI, which can help identify risk factors for poor prognosis in diabetes with AKI at an early stage and improve the short-term prognosis through timely interventions.

The results of our study showed that 54.4% of patients had stage 3 AKI, indicating that this prediction model might be more suitable for predicting the prognosis of diabetic patients with AKI in more severe AKI stages. For patients with stage 1 or 2 AKI, its predictive effect still needs to be further explored. In addition, our study showed that more severe AKI was an important risk factor for increased all-cause mortality in diabetic AKI patients (HR = 4.803). Previous studies have shown that the overall in-hospital mortality rates are 0.6% in no AKI, 5.3% in stage 1 AKI, 13.4% in stage 2 AKI, and 35.4% in stage 3 AKI (41, 42), which also supported our study. Therefore, the short-term prognosis of diabetes with AKI can be preliminarily evaluated and predicted according to the stage of AKI in clinical practice.

Previous studies have shown that advanced age is an important risk factor for the occurrence and development of patients with diabetes mellitus and AKI (43, 44). Our study also showed that advanced age was an independent risk factor for all-cause mortality in diabetic patients with AKI. A previous animal study has shown that elderly type 2 diabetes mellitus (T2DM) rats have a greater decrease in medullary blood flow and glomerular filtration rate after renal ischemia reperfusion than middle-aged T2DM rats. The expression of renal adhesion molecules and the number of infiltrating immune cells in elderly T2DM rats are higher than those in young or middle-aged rats (45). A large multicenter cohort study of 72,310 elderly patients with T2DM has shown that congestive heart failure, cerebrovascular diseases, and mortality significantly increase with increasing age (46). With age, the physiological function, self-regulation, and reserve ability of the human body decrease. In addition, the decrease in arterial wall elasticity and compliance may also aggravate vascular damage, other complications, and the incidence of clinical events in advanced-age diabetic patients with AKI.

Our study showed that a lower 25(OH)D3 was a risk factor for all-cause mortality in diabetic patients with AKI. Fernandez-Juarez et al. (47) followed up 133 patients with T2DM with proteinuria, and they reported that a low 25(OH)D3 is associated with poor prognosis (Scr increase >50%, ESRD, and mortality). A previous study has also shown a strong association between vitamin D deficiency and the increased risk of heart failure in older patients (OR = 12.19), which was similar to the result of our study (48). An animal study has shown that activation of vitamin D receptors might alleviate cisplatin-induced AKI by inhibiting iron death (49). Vitamin D3 supplementation ameliorates kidney injury induced by hyperglycemia in diabetic mice by regulating lipid metabolism, oxidative stress, apoptosis, and autophagy (50). Therefore, a lower 25(OH)D3 might contribute to all-cause mortality in diabetic patients with AKI by increasing the risk of renal damage and cardiovascular events. In addition, our study also showed that MODS was another independent risk factor for all-cause mortality in diabetic patients with AKI. A retrospective study has shown that MODS is an independent risk factor for poor prognosis in hospital-acquired AKI (OR = 3.538), which was consistent with our study (51). Hemodynamic instability and volume overload are common in patients with MODS, and the mortality of MODS is approximately 40%, which increases with the number of failing organs (52). Thus, MODS might increase the risk of all-cause mortality in diabetic patients with AKI.

### Limitations

The present study had several limitations. Firstly, the sample size was small and from only one medical center. We only verified the model internally in the same center, and the conclusions should be further confirmed by external validation at other centers. Secondly, the diagnosis of AKI in this study was only based on the criterion of Scr, which did not include the diagnostic criterion of urine volume. Thus, some patients may have been missed. Thirdly, the present study may have missed a few potential risk factors, such as microalbuminuria, albumin-to-creatinine ratio, drugs, and other therapeutic measures. Therefore, this prediction model still requires collecting more clinical data and conducting external validation in other centers to further determine its accuracy and applicability.

## Conclusion

We developed a newly generated prognostic model that has predictive value for the prognosis of diabetic patients with AKI, which has been internally verified to have good discrimination, calibration, and clinical benefit for predicting the 90-day survival rate of patients. Future studies and external validation should validate this model in different cohorts.

## Data Availability Statement

The raw data supporting the conclusions of this article will be made available by the authors, without undue reservation.

## Author Contributions

MM designed the study, analyzed data, and wrote the manuscript. LP designed the study, collected and analyzed data, and wrote the manuscript. ZH designed the study and revised the manuscript. YuL collected and analyzed data. YunL and NX collected data and revised the manuscript. All authors read and approved the final manuscript.

## Funding

This work was supported by the National Natural Science Foundation of China (no. 8176030057), the Guangxi Natural Science Foundation (no. 2018GXNSFBA050040), and the Scientific Research and Technological Development Program of Guangxi (no. GuiKeGong 1598011-6).

## Conflict of Interest

The authors declare that the research was conducted in the absence of any commercial or financial relationships that could be construed as a potential conflict of interest.

## Publisher’s Note

All claims expressed in this article are solely those of the authors and do not necessarily represent those of their affiliated organizations, or those of the publisher, the editors and the reviewers. Any product that may be evaluated in this article, or claim that may be made by its manufacturer, is not guaranteed or endorsed by the publisher.
